# Unveiling Hidden Diagnoses: The Prevalence and Clinical Impact of Extra-biliary Findings on Magnetic Resonance Cholangiopancreatography

**DOI:** 10.7759/cureus.92236

**Published:** 2025-09-13

**Authors:** Chin Kiat Tan, Mei Kei Wong, Khurram S Khan

**Affiliations:** 1 General Surgery, University Hospital Hairmyres - NHS Lanarkshire, Glasgow, GBR; 2 Diagnostic Radiology, Queen Elizabeth University Hospital, Glasgow, GBR; 3 General and Upper GI Surgery, University Hospital Hairmyres - NHS Lanarkshire, Glasgow, GBR

**Keywords:** biliary stone disease, cognitive bias, diagnostic imaging, extrabiliary findings, magnetic resonance cholangiopancreatography (mrcp)

## Abstract

Background

Magnetic resonance cholangiopancreatography (MRCP) is being increasingly employed for evaluating the biliary tree. This imaging technique has the potential to uncover extra-biliary abnormalities that may necessitate additional urgent investigations. The primary aim of this study was to determine the prevalence of extra-biliary findings on MRCPs. A secondary aim was to evaluate the subsequent need for further investigations and their outcomes.

Methods

This retrospective cohort study reviewed consecutive MRCPs performed on adult patients at a single centre between November 2020 and September 2022. Patient demographics, MRCP indications, results including extra-biliary findings, and subsequent investigations and outcomes were analysed using electronic case notes.

Results

A total of 1,018 MRCPs were included in this study; 617 (60.6%) were for female patients, and the median age was 64 years (IQR: 50-75). Specifically, 648 (63.7%) were inpatient, 418 (41.1%) were urgent, 592 (58.2%) had at least one extra-biliary finding, and 96 (9.4%) required urgent follow-up investigations, with 26 (2.6%) new diagnoses of malignancy. Additionally, 213 (20.9%) had renal cysts, 118 (11.6%) had hepatic cysts, 69 (6.7%) had pancreatic cysts, 28 (2.8%) had liver cirrhosis, 23 (2.3%) had peripancreatic collection(s), 42 (4.1%) had a hiatus hernia, 79 (7.8%) had pleural effusion(s), 46 (4.5%) had splenomegaly, and 2.6% (n=26) had identified diverticular disease.

Conclusions

Over half of the MRCPs performed identified at least one extra-biliary finding. Among those requiring urgent follow-up, over 25% led to newly diagnosed malignancies. This underscores MRCP's high sensitivity, extending beyond biliary conditions to effectively detect extra-biliary pathologies. Our findings support consideration of adopting a structured proforma for reporting MRCPs.

## Introduction

Biliary disease is one of the most common health issues in the Western world and usually presents as cholelithiasis or cholecystitis. The prevalence of cholelithiasis has been reported to be 10-15% as per the National Institute for Health and Care Excellence (NICE), UK, and other studies [1-4]. Furthermore, cholelithiasis could lead to complications such as choledocholithiasis and cholangitis, which require urgent inpatient intervention. It has been observed that approximately 10-15% of patients with cholelithiasis will have concomitant choledocholithiasis [5]. The initial investigation to confirm cholelithiasis is transabdominal ultrasonography, which boasts a high sensitivity. However, the accuracy of ultrasonography is known to be impaired by a number of factors, including operator experience, bowel gas, and poor patient compliance [6,7].

With the advancement of magnetic resonance imaging (MRI), there has been increased use of magnetic resonance cholangiopancreatography (MRCP) for biliary tree imaging. This is due to the high sensitivity of MRCPs in diagnosing choledocholithiasis as compared to transabdominal ultrasonography [8]. MRCP is regarded as the new gold standard of the biliary tree and has been advised by the NICE guidelines [9]. Furthermore, MRCP excludes the need for ionising radiation and intravenous contrast, which essentially eliminates any risk of unnecessary radiation exposure and renal dysfunction, respectively.

As MRCP images also include the lower thorax and surrounding abdomen, previous studies have shown significant incidental extra-biliary findings, some requiring further urgent investigations or treatment [10-12]. Sohns et al. showed a mean of 1.98 extra-biliary findings per patient [11], which ranged from simple cysts to new diagnoses of malignancy. This clearly demonstrated that MRCPs are underutilised in the diagnosis of extra-biliary pathology.

The primary aim of this study was to assess the prevalence of extra-biliary findings detected on MRCPs. The secondary aim was to evaluate the necessity for further investigations prompted by these findings and to analyse the clinical outcomes of such investigations. By exploring these aspects, the study aims to provide insight into the broader diagnostic value of MRCP beyond biliary pathology, potentially informing clinical decision-making and optimising patient management strategies.

This study was presented in the Association of Upper Gastrointestinal Surgery of Great Britain and Ireland (AUGIS) annual conference on September 2023 and won “Best HPB Poster Presentation” and in the Association of Surgeons of Great Britain and Ireland (ASGBI) annual conference on May 2024.

## Materials and methods

This retrospective cohort study examined consecutive MRCPs performed on adult patients at a single centre over two years, from November 2020 to October 2022, and all studies were included. The analysis involved a detailed review of various data points, including patient demographics, clinical indications for undergoing MRCP, imaging results, and specifically any extra-biliary findings observed. Data from the Scottish Index of Multiple Deprivation (SIMD) 2020 were also obtained to ascertain the degree of deprivation of patients based on their postcode. Additionally, the study evaluated the follow-up investigations prompted by these extra-biliary findings and the clinical outcomes that resulted from those investigations.

Data were obtained from electronic medical records, ensuring a comprehensive capture of patient histories, clinical presentations, and the findings on MRCPs. All MRCPs were interpreted by consultant radiologists with specialised expertise, ensuring consistency and accuracy in the reporting of both biliary and extra-biliary abnormalities. Extra-biliary findings were carefully extrapolated from the formal MRCP reports and were categorised according to their clinical relevance and whether they warranted urgent investigations based on the consultant radiologist's advice. The study also investigated the impact of these findings on patient management, specifically focusing on the subsequent diagnostic workups they prompted, such as additional imaging, biopsies, or other procedures, and the outcomes, including any new diagnoses or changes in treatment plans.

This systematic approach enabled a thorough understanding of the prevalence of extra-biliary findings and their significance in clinical practice, with the aim of informing future guidelines for reporting and managing such incidental findings in MRCPs.

This study adheres to the Strengthening the Reporting of Observational Studies in Epidemiology (STROBE) statement and checklist. This study was registered with the National Health Service (NHS) Lanarkshire’s Clinical Quality Project (project ID: 14742). Institutional review board approval was not required as this was a retrospective observational study, and the study protocols were consistent with the information governance frameworks and recommendations of NHS Scotland, national and international societies, and Caldicott requirements.

## Results

A total of 1,018 MRCPs were analysed, with patient demographics summarised in Table 1. The cohort had a predominance of female patients (60.6%), with a median age of 64 years (IQR: 50-75). Socioeconomic stratification indicated a higher proportion of patients from lower socioeconomic backgrounds. A majority (63.7%) of MRCPs were performed for inpatients, while 418 (41.1%) were categorised as urgent. The median waiting time for inpatient MRCPs was 1.2 days, significantly shorter than the 26.9-day median for outpatient imaging.

**Table 1:** Demographics of the patients included in the study SIMD: Scottish Index of Multiple Deprivation

Patient Categories	Number of Patients
Male	401 (39.4%)
Female	617 (60.6%)
Median Age	64 years (IQR: 50-75)
Ethnicity (White Scottish)	993 (97.5%)
SIMD 1^st^ quantile	195 (19.2%)
SIMD 2^nd^ quantile	274 (26.9%)
SIMD 3^rd^ quantile	226 (22.2%)
SIMD 4th quantile	153 (15.0%)
SIMD 5^th^ quantile	166 (16.3%)
Unrecognised postcode	4 (0.4%)

**Figure 1:**
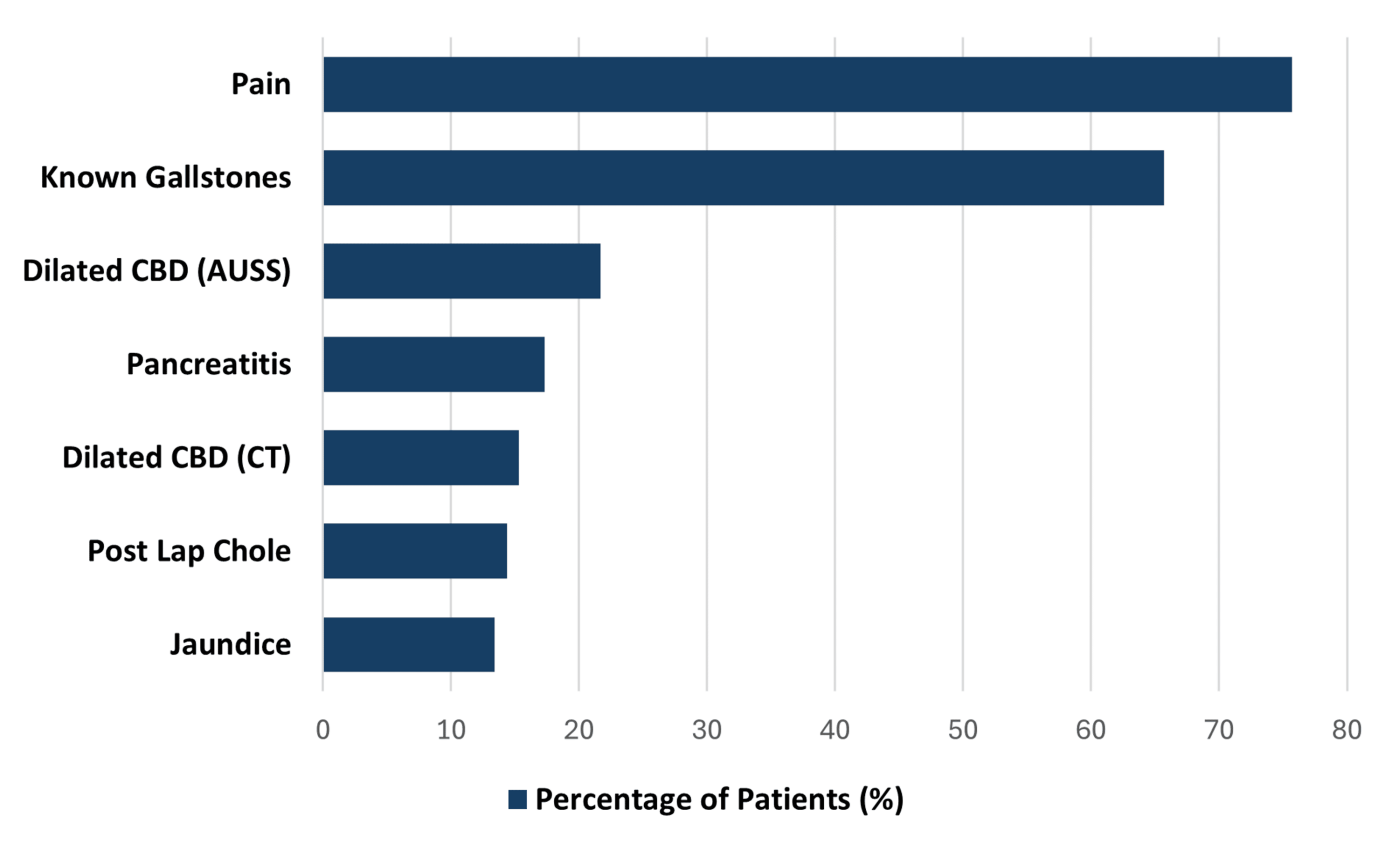
Breakdown of the indications for a n MRCP request Each MRCP request was scrutinised and noted to possibly contain multiple indications. CBD: Common bile duct; MRCP: Magnetic resonance cholangiopancreatography

The primary clinical indications for MRCP included right upper quadrant or epigastric pain (n=771, 75.7%) and known cholelithiasis (n=669, 65.7%), as illustrated in Figure 1. Notably, a substantial proportion of patients had multiple indications for undergoing the scan.

**Figure 2:**
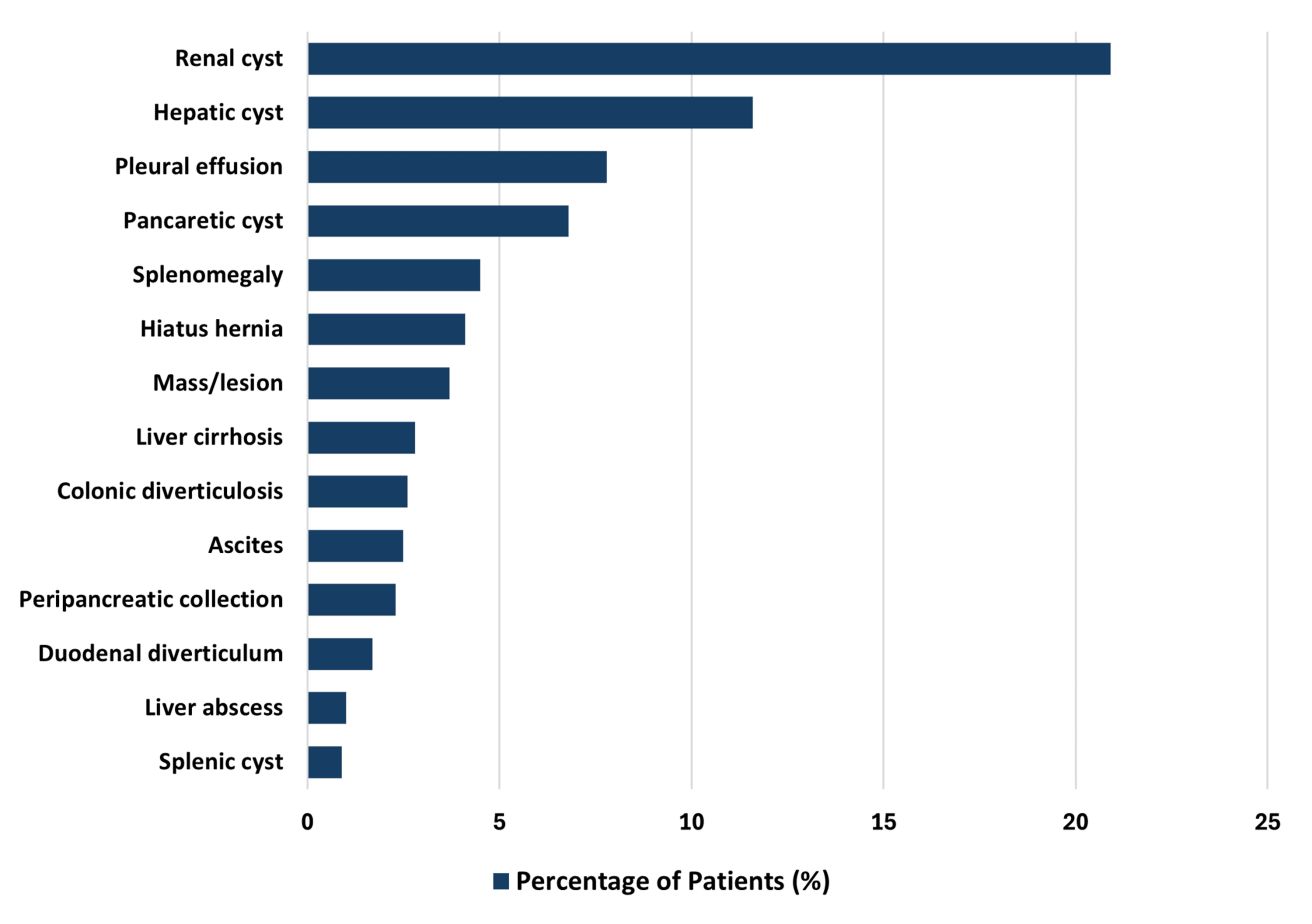
Breakdown of extra-biliary findings Magnetic resonance cholangiopancreatography (MRCP) reports can contain more than one extra-biliary finding.

Extra-biliary findings were detected in 592 cases (58.2%), with 96 (9.4%) necessitating urgent follow-up and 496 (48.8%) requiring non-urgent surveillance. Among patients who underwent further evaluation, 26 (2.6%) were diagnosed with malignancies. The most frequently encountered extra-biliary abnormalities included renal cysts (n=213, 20.9%), hepatic cysts (n=118, 11.6%), pleural effusion (n=79, 7.8%), and pancreatic cysts (n=69, 6.7%). Less common but clinically significant findings included liver cirrhosis (n=28, 2.8%), peripancreatic collections (n=23, 2.3%), and splenomegaly (n=46, 4.5%) (Figure 2).

**Figure 3:**
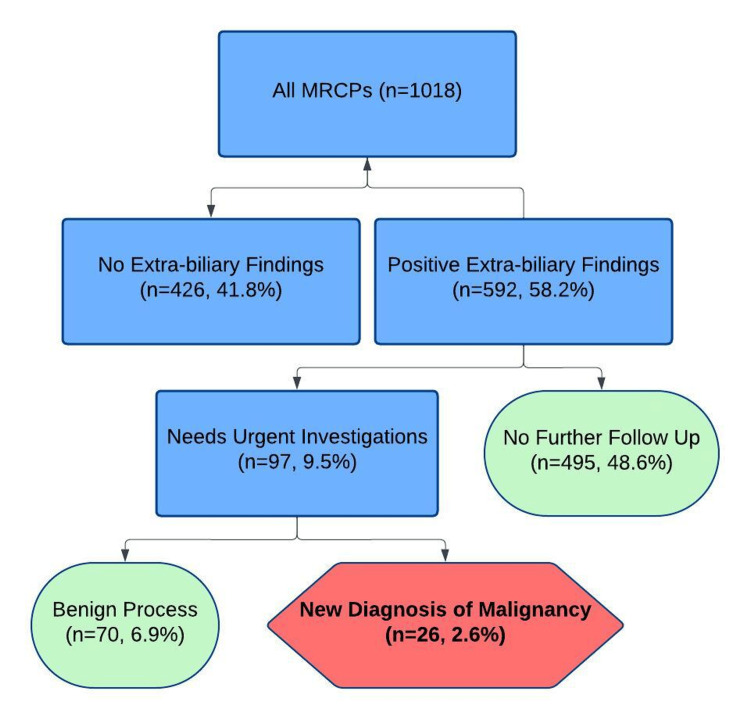
Pathway for investigating the extra-biliary findings MRCP: Magnetic resonance cholangiopancreatography

Of the 96 patients requiring urgent follow-up, subsequent investigations included cross-sectional imaging, endoscopic evaluation, or biopsy. Malignancies were confirmed in 26 cases (4.4% of those with extra-biliary findings), underscoring the clinical relevance of these incidental diagnoses. The pathways for evaluating and managing extra-biliary findings are outlined in Figure 3.

## Discussion

This study demonstrates that a significant proportion of MRCP examinations reveal extra-biliary findings, with more than half of the cases (58.2%) including at least one such finding. Notably, 9.4% of these findings warranted urgent follow-up investigations, with 2.6% leading to new malignancy diagnoses. These results underscore the broader diagnostic utility of MRCP beyond its primary role in evaluating biliary pathology and highlight the potential clinical impact of incidental findings on patient management and outcomes, which agrees with previous studies.

The ability of MRCPs to detect extra-biliary abnormalities is largely attributed to their comprehensive imaging coverage, encompassing the liver, pancreas, kidneys, and portions of the gastrointestinal tract. To our knowledge, there have been a limited number of studies conducted to explore the extent of extra-biliary findings on MRCPs. Our findings align with prior studies that have identified a significant rate of extra-biliary incidentalomas, including renal, hepatic, and pancreatic cysts, liver cirrhosis, peripancreatic collections, pleural effusions, and splenomegaly [10-12]. While many of these findings are benign, a subset represents clinically significant pathology that necessitates further diagnostic evaluation and intervention.

Despite the clear diagnostic advantages, there remains a degree of variability in how radiologists approach and report extra-biliary findings on MRCPs, reflecting potential interobserver variability. Previous literature has suggested that radiologists may not consistently document incidental findings due to factors such as perceptual errors, cognitive biases, or concerns regarding the potential for overdiagnosis and unnecessary investigations [13]. Research indicates that approximately 80% of radiology reporting errors are perceptual in nature [14], emphasising the need for standardised protocols to ensure comprehensive and systematic assessment of all relevant anatomical structures.

One of the longstanding concerns regarding MRCPs has been their cost-effectiveness compared to other imaging modalities. However, economic analyses have demonstrated that MRCP provides substantial cost benefits by improving patient selection for invasive procedures such as endoscopic retrograde cholangiopancreatography (ERCP). By reducing the rate of unnecessary ERCPs, MRCP minimises the risks associated with invasive interventions, including procedural complications and hospital admissions, further justifying its routine use in biliary disease evaluation [15,16].

Given the clinical implications of extra-biliary findings, there is a strong argument for implementing structured reporting systems in MRCP interpretation. A standardised proforma could facilitate consistent documentation of extra-biliary abnormalities, reduce the likelihood of missed diagnoses, and support timely clinical decision-making. It has been shown in previous studies that standardised reporting for both benign and malignant pathologies has improved the quality of reports and aids in decision making [17-19]. Furthermore, multidisciplinary collaboration between radiologists, hepatobiliary surgeons, and gastroenterologists could enhance the management of incidental findings, ensuring appropriate follow-up strategies tailored to the clinical significance of each abnormality.

While this study provides valuable insights into the prevalence and impact of extra-biliary findings on MRCP, certain limitations must be acknowledged. The retrospective nature of the analysis may introduce selection bias, and the study was conducted within a single centre, potentially limiting the generalisability of the findings. Additionally, the study period coincided with the COVID-19 pandemic, which may have influenced imaging practices, patient prioritisation, and radiological reporting trends. Future multi-centre prospective studies would be beneficial in further validating these findings and exploring the long-term clinical outcomes associated with incidental extra-biliary abnormalities on MRCP.

## Conclusions

MRCP provides extensive diagnostic information due to its broad anatomical coverage, often revealing significant extra-biliary findings. Given the high prevalence of biliary disease and the frequent utilisation of MRCP for biliary assessment, the implementation of standardised reporting protocols may enhance the detection of clinically relevant extra-biliary pathologies, including malignancies. Incorporating a systematic approach to MRCP interpretation could reduce the likelihood of missed diagnoses and improve patient outcomes.
